# The Heme Transport Capacity of LHR1 Determines the Extent of Virulence in *Leishmania amazonensis*

**DOI:** 10.1371/journal.pntd.0003804

**Published:** 2015-05-22

**Authors:** Rebecca L. Renberg, Xiaojing Yuan, Tamika K. Samuel, Danilo C. Miguel, Iqbal Hamza, Norma W. Andrews, Andrew R. Flannery

**Affiliations:** 1 Department of Cell Biology and Molecular Genetics, University of Maryland, College Park, Maryland, United States of America; 2 Department of Animal and Avian Sciences, University of Maryland, College Park, Maryland, United States of America; 3 PathSensors, Inc., Baltimore, Maryland, United States of America; McGill University, CANADA

## Abstract

*Leishmania* spp. are trypanosomatid parasites that replicate intracellularly in macrophages, causing serious human morbidity and mortality throughout the world. Trypanosomatid protozoa cannot synthesize heme, so must acquire this essential cofactor from their environment. Earlier studies identified LHR1 as a *Leishmania amazonensis* transmembrane protein that mediates heme uptake. Null mutants of LHR1 are not viable and single knockout strains have reduced virulence, but very little is known about the properties of LHR1 directly associated with heme transport. Here, we use functional assays in *Saccharomyces cerevisiae* to show that specific tyrosine residues within the first three predicted transmembrane domains of LHR1 are required for efficient heme uptake. These tyrosines are unique to LHR1, consistent with the low similarity between LHR1 and its corresponding homologs in *C*. *elegans* and human. Substitution of these tyrosines in LHR1 resulted in varying degrees of heme transport inhibition, phenotypes that closely mirrored the impaired ability of *L*. *amazonensis* to replicate as intracellular amastigotes in macrophages and generate cutaneous lesions in mice. Taken together, our results imply that the mechanism for heme transport by LHR1 is distinctive and may have adapted to secure heme, a limiting cofactor, inside the host. Since LHR1 is significantly divergent from the human heme transporter HRG1, our findings lay the groundwork for selective targeting of LHR1 by small molecule antagonists.

## Author Summary

*Leishmania* are protozoan parasites that infect humans and replicate intracellularly in macrophages, cells normally engaged in protecting the host from pathogens. These parasites have several strategies to survive inside the hostile environment of the host macrophage, and one of these strategies involves heme acquisition. Heme is an iron-containing molecule that is essential for many cellular functions. Unlike mammalian cells, *Leishmania* parasites cannot synthesize heme, so must acquire it from the host cell. In earlier work we found that the parasites express a surface protein, LHR1, which transports heme into the parasites. In this study we identified specific amino acids in LHR1 that are required for heme transport. When expressed in yeast cells, LHR1 carrying these mutations had defects in heme transport that were equivalent to the inhibition in virulence observed when these proteins were expressed in *Leishmania* and tested in macrophage and mouse infection assays. These critical amino acids do not exist in the human heme transporter, indicating that LHR1 is a promising target for the development of specific drugs for the treatment of leishmaniasis and possibly other serious parasitic diseases, such as Chagas’ disease and sleeping sickness.

## Introduction

*Leishmania* spp. are protozoan parasites from the Trypanosomatidae family that cause leishmaniasis, a wide-spectrum disease that ranges from self-healing cutaneous lesions to lethal visceralizing infections. With more than twenty known species of *Leishmania* that can cause disease in humans, leishmaniasis is estimated to be the ninth largest infectious disease burden in the world, with an estimated 1.3 million new infections reported each year [1]. Current treatments are expensive, toxic, and are gradually becoming ineffective with the rise of drug resistance in endemic areas [2, 3]. Consequently, there is a great need for the development of new drugs that are more affordable, less toxic, and that have greater efficacy against the disease.

*Leishmania* has a bimodal life cycle, alternating between a sand fly vector and a vertebrate host. In vertebrates *Leishmania* is an intracellular parasite of macrophages, replicating within parasitophorous vacuoles (PV), which have properties similar to lysosomes. Nutrient availability within the PV is known to have drastic effects on parasite growth and disease outcome [4, 5]. Two of these essential nutrients, iron and heme, are in limited supply in late endocytic compartments [6]. Iron is a critical element for many biological processes because its oxidation-reduction potential facilitates multiple types of electron transfer reactions. Both host and parasite require iron to perform essential biological functions, and the ability of hosts to limit the access of pathogens to iron is an effective mechanism for controlling infections [7, 8]. This “battle for iron” between host and pathogen is illustrated well by the role of the Natural resistance associated macrophage protein 1(Nramp1) transporter, which removes iron and other divalent cations from late endocytic compartments of macrophages [9–11] and is a host susceptibility gene for *Leishmania* infection [12]. In *L*. *amazonensis*, the ferric iron reductase LFR1 and the ferrous iron transporter LIT1 work in concert to promote iron uptake and parasite virulence [6, 13–15]. More recently, *Leishmania* parasites were also shown to influence the ability of the host cell to regulate iron pools, by stimulating iron uptake and inhibiting iron export by macrophages [16, 17].

Heme is an iron-containing porphyrin that also plays a central role in iron availability at the host-parasite interface, via the recycling of iron during erythrophagocytosis by macrophages [18, 19]. Heme functions as an essential prosthetic group for many enzymes, involved in a variety of critical cellular functions [20]. *Leishmania* and other trypanosomatid protozoa are heme auxotrophs that lack the first five enzymes in the heme biosynthetic pathway; to survive these parasites must acquire heme from the environment [6, 21, 22]. *L*. *amazonensis* was shown to bind heme specifically more than two decades ago [23], but the molecule(s) responsible for heme transport in these parasites remained elusive until more recently. The *L*. *donovani* ATP-Binding Cassette G5 (LABCG5) was proposed to be involved in heme salvage after breakdown of hemoglobin, but not in direct heme uptake from the environment [24]. Studies in *L*. *amazonensis* identified the transmembrane protein LHR1, that promotes heme transport across the membrane [25] and is required for parasite virulence in macrophage and mouse infections [26]. The results of these studies emphasized the essential role played by heme availability in the replication and intracellular survival of *Leishmania*, and highlighted the need for a better understanding of the process by which these parasites acquire, traffic, and store heme. Syntenic genes with high sequence identity to *L*. *amazonensis* LHR1 are present in the genomes of several pathogenic trypanosomatid parasites, making this transporter a potentially promising target for the development of new drugs to treat a number of serious tropical diseases [27]. However, to achieve this goal it is first necessary to understand unique features of LHR1 that regulate heme uptake, and to directly correlate these features with parasite virulence. In this study we identify unique transmembrane tyrosines that regulate heme transport by *Leishmania* LHR1, and show that mutations in these amino acids modulate the extent of heme uptake and the virulence of *L*. *amazonensis*.

## Methods

### Sequence Alignment

*C*. *elegans HRG-4* sequence was provided by Dr. Iqbal Hamza (NCBI Reference Sequence: NM_001136385.1), *L*. *amazonensis LHR1* sequence was performed in the Andrews’ lab, *L*. *major* and *L*. *infantum LHR1* sequences were downloaded from TriTrypDB (LmjF.24.2230 and LinJ.24.2320). Sequences were aligned using Clustal Omega (http://www.ebi.ac.uk/Tools/msa/clustalo/). Transmembrane domain predictions were performed using TMHMM 2.0 (http://www.cbs.dtu.dk/services/TMHMM-2.0/).

### Yeast Strains and Growth Medium

The *Saccharomyces cerevisiae* strain Δ*hem1* (6D) strain was constructed as described elsewhere [28]. Cells were maintained in YPD (yeast extract-peptone-dextrose) or appropriate synthetic complete (SC) medium supplemented with 250 μM δ-aminolevulinic acid hydrochloride (ALA) (Frontier Scientific; Cat. #A167). *Saccharomyces cerevisiae* W303 was maintained in YPD or appropriate SC medium.

### Plasmid Constructs and Site-Directed Mutagenesis in Yeast

pYesDEST52-LHR1-HA was constructed as described elsewhere [25]. Site-directed mutagenesis was performed on pYesDEST52-LHR1-HA using the QuikChange Site-Directed Mutagenesis protocol (Agilent Technologies; Cat. # 200518); Table 1 below describes the primers used.

**Table 1:** 

Y18A	
F	5ʹ-CCGCATTTGGTTGGCGATCGCCATAATATTCGCCCTGATC-3ʹ
R	5ʹ-GATCAGGGCGAATATTATGGCGATCGCCAACCAAATGCGG-3ʹ
H36A	
F	5ʹ-TATTACTTTTTTGCTTTGTGGCCCAGAACTACTGGGCCTGTG-3ʹ
R	5ʹ-CACAGGCCCAGTAGTTCTGGGCCACAAAGCAAAAAAGTAATA-3ʹ
Y64A	
F	5ʹ-GGCGACGCCGCAGGCCCGCCTGTACAAG-3ʹ
R	5ʹ-CTTGTACAGGCGGGCCTGCGGCGTCGCC-3ʹ
Y80A	
F	5ʹ-TGCAGCTTCAACCGCATTGCAGCCCAGACGTTCTTTCTTTG-3ʹ
R	5ʹ-CAAAGAAAGAACGTCTGGGCTGCAATGCGGTTGAAGCTGCA-3ʹ
H105A	
F	5ʹ-GGCGAGGGGCATTAAGGCTCGCCAGTCTTGG-3ʹ
R	5ʹ-CCAAGACTGGCGACGCTTAATGCCCCTCGCC-3ʹ
R106A	
F	5ʹ-GCGAGGGGCATTAAGCATGCCCAGTCTTGGACAG-3ʹ
R	5ʹ-CTGTCCAAGACTGGGCATGCTTAATGCCCCTCGC-3ʹ
Y129A	
F	5ʹ-CGGCGAAGTGGAGCGCCATCGTGACGGTGC-3ʹ
R	5ʹ-GCACCGTCACGATGGCGCTCCACTTCGCCG-3ʹ
Y136A	
F	5ʹ-TGACGGTGCCCATCGCCGAGCTCCGAGAGG-3ʹ
R	5ʹ-CCTCTCGGAGCTCGGCGATGGGCACCGTCA-3ʹ
C174A	
F	5ʹ-GCGATGTCAAAGGAGAACGCTGCATACCCATACGACGT-3ʹ
R	5ʹ-ACGTCGTAT GGGTATGCAGCGTTCTCCTTTGACATCGC-3ʹ

To generate yeast expression plasmids for the glycerol/lactate spot growth assay, pYesDEST52-yLHR1-HA WT, Y18A, H36A, Y80A or Y129A plasmids were amplified by PCR using gene specific primers (5-BglII-yLHR1: 5ʹ-GACCGCGAGATCTAAAAAAATGAACGAATT AGAAAGAAAG-3ʹ, 3-XhoI-yHA: 5ʹ-GGACTGACATCTCGAGTTAAGCATAATCA GGAACATCGTATGGGTA-3ʹ), digested with BglII and XhoI, and ligated into Yep352/PGK91-2 vector (Gift from Dr. Caroline C. Philpott).

To generate yeast expression plasmids for the Gallium (III) Protoporphyrin IX (GaPPIX) spot growth assay, the yeast codon-optimized LHR1 (pYesDEST52-yLHR1) that was previously described [25] was tagged at the c-terminus with the HA epitope using primers 5ʹ-CGTCGTATGGGTAACCTGCACAGTTTT CCTTTG-3ʹ and 5ʹ-TCCCAGACTACGCTTAATCTAGAGGGCCCTTC-3ʹ to generate pYesDEST52-yLHR1-HA. pYesDEST52-yLHR1-HA was then used to generate the mutants using the QuikChange Site-Directed Mutagenesis protocol; Table 2 below describes the primers used.

**Table 2:** 

yLHR1-HA Y18A	
F	5ʹ-CAAATATATCAAGGCGAAGATGATAGCGATTGCCAACCAGATTCTAAAAGTT-3ʹ
R	5ʹ-AACTTTTAGAATCTGGTTGGCAATCGCTATCATCTTCGCCTTGATATATTTG-3ʹ
yLHR1-HA H36A	
F	5ʹ-GCAAGCCCAATAGTTTTGAGCTACGAAACAAAATAACAAAACACCAGACAAAT-3ʹ
R	5ʹ-ATTTGTCTGGTGTTTTGTTATTTTGTTTCGTAGCTCAAAACTATTGGGCTTGC-3ʹ
yLHR1-HA Y80A	
F	5ʹ-AAGCACACAAGAAAAAGGTTTGGGCGGCGATTCTATTGAAAGAACAAG-3ʹ
R	5ʹ-CTTGTTCTTTCAATAGAATCGCCGCCCAAACCTTTTTCTTGTGTGCTT-3ʹ
yLHR1-HA Y129A	
F	5ʹ-GATTGGGACGGTTACTATAGCGGACCACTTTGCAGCCATC-3ʹ
R	5ʹ-GATGGCTGCAAAGTGGTCCGCTATAGTAACCGTCCCAATC-3ʹ

### Spot Growth Assay

pYesDEST52-LHR1-HA, pYesDEST52-LHR1-HA-Y18A/H36A/Y64A/Y80A/H105A/R106A/Y129A/Y136A/C174A, or vector alone (pYesDEST52) were transfected into Δ*hem1* (6D) using the lithium acetate method [29]. Spotting of cultures was performed as described previously [30] with some adaptation. Briefly, transformants were selected on 2% w/v glucose SC (-Ura) plates supplemented with 250 μM ALA. Five to seven colonies were picked and inoculated into 2% w/v raffinose SC (-Ura) supplemented with 250 μM ALA to deplete glucose and expand the culture, then subcultured in 2% w/v raffinose SC (-Ura) and grown overnight to deplete heme. Cultures were resuspended in water to OD_600_ of 0.2 and ten-fold serial dilutions were performed; 10 μl of each dilution was spotted onto a 2% w/v raffinose SC (-Ura) plate supplemented with either 0.4% w/v glucose plus 250 μM ALA (positive control) or 0.4% w/v galactose plus a range of heme concentrations (no heme added as negative control). Plates were incubated at 30°C and imaged on day four.

### β-Galactosidase Reporter Assay

pYesDEST52-LHR1-HA [25] and pRS314m-CYC1-LacZ [30] were constructed as described elsewhere. This assay was performed as described previously [25, 30]. Briefly, the plasmids for expression of LHR1 wild type or mutant proteins (pYesDEST52-LHR1-HA or mutants) or vector alone (pYesDEST52) was co-transformed with the β-galactosidase reporter construct (pRS314m-CYC1-LacZ) into Δ*hem1* (6D) strain using the lithium acetate method [29] and transformants were selected on 2% w/v glucose SC (-Ura-Trp) supplemented with 250 μM ALA. Five to seven colonies were picked from the selection plates and inoculated into 2% w/v raffinose SC (-Ura-Trp) supplemented with 250 μM ALA, subcultured in 2% w/v raffinose SC (-Ura-Trp) and incubated overnight at 30°C to deplete heme. Heme depleted cultures were then used to inoculate 2% w/v raffinose SC (-Ura-Trp) supplemented with either 0.4% w/v glucose plus 250 μM ALA (positive control) or 0.4% w/v galactose plus a range of heme concentrations (no heme added for negative control) at an OD_600_ of 0.1 and incubated at 30°C, 250 rpm for 20 h. Cells were resuspended in breaking buffer (100 mM Tris-HCl pH 8, 1 mM dithiothreitol (DTT), 20% glycerol) with 1X cOmplete protease inhibitor cocktail (Roche Cat. # 04693159001) with an equal volume of 0.5 mm glass beads (Sigma Cat. # G8772) and disrupted using a bead beater for 1 min, 3 times at 4°C. β-galactosidase activity was determined in the cell lysates using the assay as described elsewhere [31]. β-galactosidase activities were normalized to protein concentration for direct comparison.

### Immunoblotting of Yeast Protein Extracts

Cells were resuspended in Thorner buffer with an equal volume of glass beads (Sigma Cat. # G8772) and vortexed for 10 min at room temperature. Equal amounts of protein were loaded into each lane and run on a 12% SDS-PAGE gel and transferred to a nitrocellulose membrane (BioRad; Cat. #162–0115). Membranes were blocked with 5% milk, incubated with rabbit anti-HA (AbCam; Cat. # ab9110) at 1:4,000 or mouse anti-Vma2 (Life Technologies; Cat.# A-6427) at 1:4000, followed by HRP-conjugated donkey anti-rabbit IgG (Jackson ImmunoResearch Labs; Cat. # 711-035-152) at 1:10,000 or HRP-conjugated donkey anti-mouse (Jackson ImmunoResearch Labs; Cat. # 715-035-151) at 1:10,000. Membranes were developed using Clarity Western ECL Substrate (BioRad; Cat. # 170–5060) and imaged using a FujiFilm LAS-3000.

### Glycerol/Lactate Spot Growth Assay

The plasmids for yLHR1 expression (Yep352/PGK91-2-yLHR1-HA WT/Y18A/H36A/Y80A/Y129A) or vector alone (Yep352/PGK91-2) were transformed into strain Δ*hem1* (6D) using the lithium acetate method [29]. Transformants were selected on 2% w/v glucose SC (-Ura) plates supplemented with 250 μM ALA. Five or six transformed colonies were picked and streaked on 2% w/v raffinose SC (-Ura) plates supplemented with 250 μM ALA to deplete glucose for 48 h. Prior to spotting, cells were cultivated in 2% w/v glycerol/lactate SC (-Ura) medium for 18 h to deplete heme. Cells were then suspended in water to an OD_600_ of 0.2 and ten-fold serial dilutions of each transformant was spotted (10 μl /spot) onto 2% w/v glycerol, 2% w/v lactate SC (-Ura) plates supplemented with either 250 μM ALA (positive control), or a range of concentrations of heme and incubated at 30°C for four days before imaging.

### GaPPIX Spot Growth Assay

The plasmids for yLHR1 expression (pYesDEST52-yLHR1-HA WT/Y18A/H36A/Y80A/Y129A) or vector along (pYesDEST52) were transformed into *S*. *cerevisiae* strain W303 using the lithium acetate method [29]. Yeast transformants were selected on 2% w/v glucose SC (-Ura) plates. Five or six transformed colonies were picked, streaked and grown on 2% w/v raffinose SC (-Ura) plates for 48 h. Cells were then cultivated in 2% w/v raffinose SC (-Ura) medium for 18 h, suspended in water to an OD_600_ of 0.2, and ten-fold serial dilutions were performed. Serial dilutions were spotted (10 μl /spot) onto 2% w/v raffinose, 0.4% w/v galactose SC (-Ura) plates supplemented with a range of GaPPIX concentrations, incubated at 30°C for 3 days, and imaged. 10mM GaPPIX (Frontier Scientific, Cat. # P40167) stock solution was prepared by dissolving GaPPIX in 0.3N NH_4_OH, adjusting pH to 8.0 with concentrated HCl.

### Parasite Cultures

*L*. *amazonensis* IFLA/BR/67/PH8 strain was provided by Dr. David Sacks (Laboratory of Parasitic Diseases, NIAID, NIH). *L*. *amazonensis* Δ*lhr1/LHR1* (SKO) was generated as previously described [25]. Parasites were cultured at 26°C in promastigote growth medium (PGM): M199 (Gibco BRL; Cat. #11825–015) pH 7.4 supplemented with 20% heat-inactivated FBS, 40 mM HEPES pH 7, 0.1 mM adenine, 0.00001% biotin, 0.0005% hemin (7.6 μM), 5 mM L-Glutamine, and 5% penicillin-streptomycin. Amastigotes were generated axenically by mixing 1:1 a late-log promastigote culture with amastigote growth media (AGM): M199 pH 4.5 supplemented with 20% heat-inactivated FBS, 0.00025% hemin (3.8 μM), 20 mM succinate buffer pH 4.5, 0.25% glucose, 0.5% trypticase, 0.1 mM adenine, 5 mM L-Glutamine, and 5% penicillin-streptomycin. Cultures were maintained at 26°C overnight and then transferred to 32°C for three to four days after which they were split 1:2 and incubated at 32°C for an additional three to four days and used on day seven or eight for infections in macrophages or mice.

### *Leishmania* Expression Plasmids

Cloning of *LHR1-HA* WT/Y18A/H36A/Y80A/ Y129A into *Leishmania* expression vector pXG-SAT (courtesy of Dr. S. Beverley, Washington University) was done using the In-Fusion HD Cloning System (Clontech; Cat. # 639645) following the manufacturer’s instructions. Briefly, pYesDEST52-*LHR1-HA* WT, Y18A, H36A, Y80A or Y129A plasmids were used to amplify the ORF using primers designed for In-Fusion Cloning; Table 3 below describes the primers used. The PCR fragment was then sub-cloned into pXG-SAT that had been linearized by BamHI.

**Table 3:** 

In-Fusion Cloning Forward	In-Fusion Cloning Reverse
5ʹ-TGTCCCCGGGGGATCCATGAACGAGTTGGAGCGCAA-3ʹ	5ʹ-ATCTGCTAGTGGATCCTTAAGCGTAGTCTGGGACGT-3ʹ

### *Leishmania* Transfections

pXGSAT-LHR1-HA WT/Y18A/H36A/Y80A/Y129A were transfected into *L*. *amazonensis* Δ*lhr1/LHR1* as previously described [32]. Parasites resistant to 50 μg/ml nourseothricin were selected and screened for expression using immunoblotting.

### Macrophage Infections

C57BL/6 mouse bone marrow macrophages (BMM) were obtained as previously described [33]. Approximately 1x10^6^ BMM were plated on glass coverslips that had been placed in a 6-well plate using RPMI medium (Invitrogen/Gibco; Cat. # 11875–119) supplemented with 20% endotoxin-free FBS, 1% penicillin/streptomycin, 2 mM L-glutamine, 50 ng/ml human recombinant human macrophage colony-stimulating factor (M-CSF) (PeproTech; Cat. # 300–25) and incubated for 24 h at 37°C, 5% CO_2_. BMMs were infected using axenic amastigotes at a ratio of two parasites per BMM for 4 h at 34°C. The cells were washed 3 times with PBS and further incubated for 0, 36,or 72 h at 34°C. Coverslips were fixed in 4% paraformaldehyde and permeabilized with 0.1% Triton X-100 for 10 min. BMM and *Leishmania* DNA were stained with 1 μg/ml DAPI (4’,6-diamidino-2-phenylindole) and washed extensively with PBS and mounted using ProLong Gold Antifade (LifeTechnologies; Cat. # P36934). The number of intracellular parasites was determined by counting the total macrophages and the total intracellular parasites per microscopic field using a Nikon E200 epifluorescence microscope. Counts were performed in triplicate for each period of infection. The number of amastigotes was divided by the number of macrophages and multiplied by 100 to obtain number of amastigotes per 100 BMM.

### Immunofluorescence

For *S*. *cerevisiae*, plasmids for yLHR1 expression (pYesDEST52-yLHR1-HA WT/Y18A/H36A/Y80A/Y129A) or vector alone (pYesDEST52) were transformed into the Δ*hem1* (6D) strain, selected for as described above, then grown in 2% w/v raffinose, 0.4% w/v galactose SC (-Ura) supplemented with 250 μM ALA starting at an OD_600_ of 0.1 until an OD_600_ of 0.6–0.8. The cells were then fixed in 4% formaldehyde for 1 h at 30°C with shaking, resuspended in 4% PFA supplemented with 100 mM KH_2_PO_4_ and incubated overnight with shaking at room temperature. Cells were then resuspended in TEβ (200 mM Tris pH 8.0, 20 mM EDTA, 1% β-mercaptoethanol), incubated at 30°C for 10 min, pelleted and resuspended in spheroplast solution (1.2 M sorbitol, 50 mM KH_2_PO_4_ pH 7.4, 1 mM MgCl_2_, 0.1 U/ul zymolyase (Zymo Research; Cat. # E1004)) followed by incubation at 30°C for 45 min and washes in 1.2 M sorbitol. Spheroplasts were permeabilized in 0.6 M sorbitol with 1% SDS for 2 min, washed with 1.2 M sorbitol and attached to coverslips coated with Poly-L-Lysine (Sigma Aldrich; Cat. # P8920). After blocking for 1 h with 1% horse serum in PBS-BSA (PBS supplemented with 5 mg/ml BSA) spheroplasts were incubated with 1:1000 anti-HA (BioLegend; Cat. # MMS-101P) antibodies in PBS-BSA for 2 h, followed by Alexa Fluor 488 goat anti-mouse (Invitrogen; Cat. # A11001) at 1:10,000 in PBS-BSA for 1 h and mounted using ProLong Gold Antifade. Images were acquired using a DeltaVision Elite Deconvolution Microscope (GE Healthcare) under identical acquisition settings.

For *Leishmania*, C57BL/6 mouse BMM were plated on coverslips and infected using axenic *L*. *amazonensis* amastigotes as described above. Infected BMM were fixed 24 h post infection with 4% paraformaldehyde, quenched with 15 mM NH_4_Cl, permeabilized with 0.5% saponin in PBS +/+, and blocked with 5% horse serum in SBP (PBS +/+ containing 0.1% saponin and 1% BSA). The coverslips were then stained with mouse anti-HA (HA.11 Clone 16B12) (BioLegend; Cat. # MMS-101P) diluted 1:1,000 in SBP and anti-BiP (gift from Dr. J.D. Bangs, University at Buffalo School of Medicine and Biomedical Sciences) diluted 1:1,000 in SBP. Cells were washed with SBP followed by staining with Alexa Fluor 488 goat anti-mouse IgG (Invitrogen; Cat. # A11001) diluted 1:1,000 in SBP and Alexa Fluor 647 donkey anti-rabbit IgG (Invitrogen; Cat. # A31573) diluted 1:1,000 in SBP followed by washing with SBP and PBS +/+. Cells were stained with 1 μg/ml DAPI and washed extensively with PBS +/+. Coverslips were mounted using ProLong Gold Antifade and images were acquired using a DeltaVision Elite Deconvolution Microscope (GE Healthcare) under similar acquisition settings.

### Mouse Infections

Six-week-old female C57BL/6 mice (*n* = 5 per group) were inoculated with 1x10^6^ axenic amastigotes of wild type *L*. *amazonensis* (Lam), Δ*lhr1/LHR1* (SKO), Δ*lhr1/LHR1* + *LHR1-HA* (SKO+WT), Δ*lhr1/LHR1* + *LHR1-HA-Y18A* (SKO+Y18A), Δ*lhr1/LHR1* + *LHR1-HA-H36A* (SKO+H36A), Δ*lhr1/LHR1* + *LHR1-HA-Y80A* (SKO+Y80A), or Δ*lhr1/LHR1* + *LHR1-HA-Y129A* (SKO+Y129A) *L*. *amazonensis* in the left hind footpad in a volume of 50 μl sterile PBS. Lesion (non-ulcerated) progression was monitored once a week by measuring the thickness of the left and right hind footpads with a caliper (Mitutoyo Corp., Japan), and expressing the results as the difference between the two. The parasite burden recovered from the infected tissue was determined after ten weeks by limiting dilution as described elsewhere [34].

### Ethics Statement

This study was performed in strict accordance with the recommendations in the Guide for the Care and Use of Laboratory Animals of the National Institutes of Health. The Institutional Animal Care and Use Committee (IACUC) of the University of Maryland, College Park approved the animal study protocol (IACUC protocol number: R-14-79).

## Results

**Fig 1:**
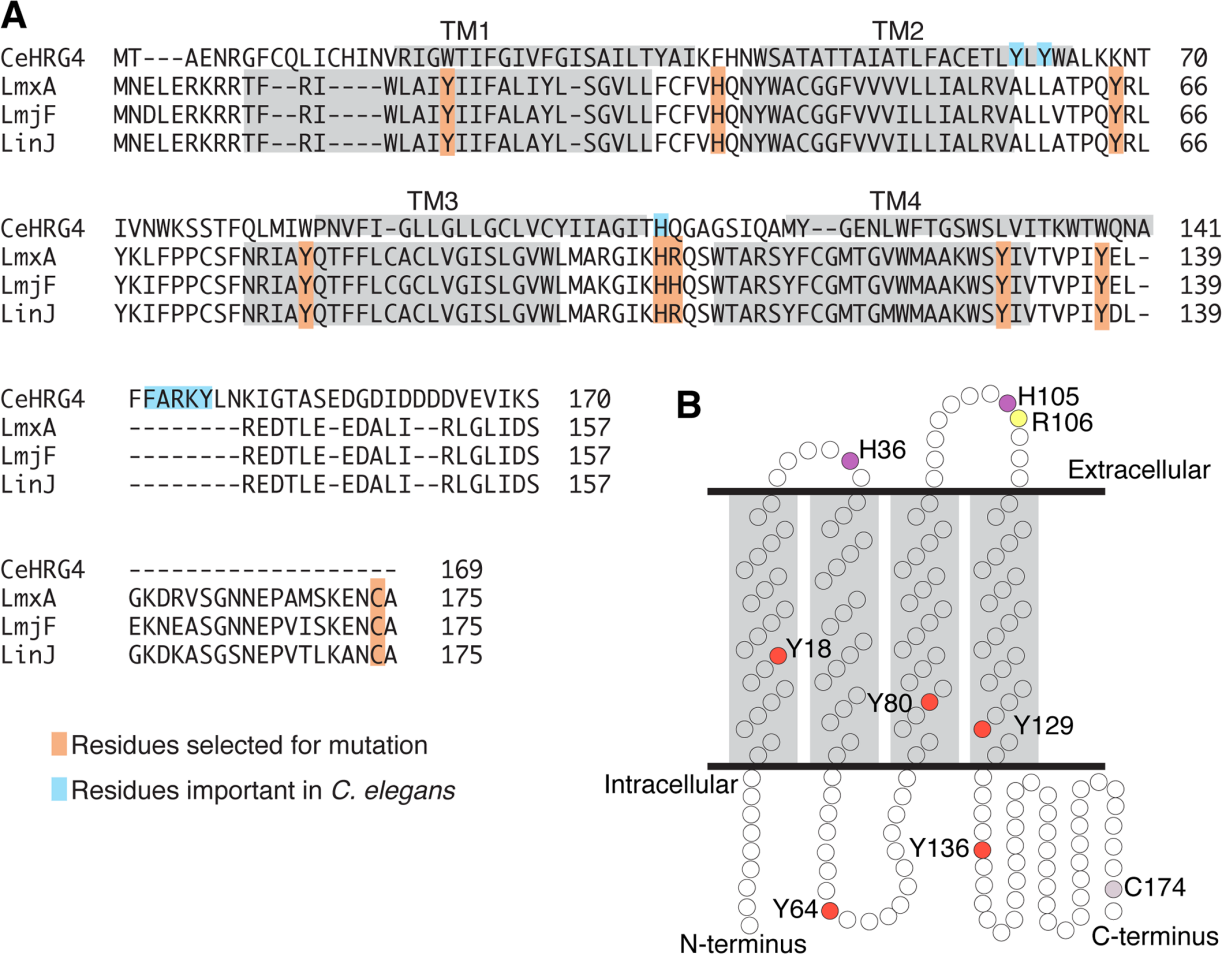
Amino acid residues targeted for mutagenesis in *L*. *amazonensis* LHR1. **A**) Alignment of *C*. *elegans* CeHRG-4 with *L*. *amazonensis* (LmxA) LHR1 and LHR1 homologues from *L*. *major* (LmjF) and *L*. *infantum* (LinJ) highlighting the residues selected for mutation analysis. Light blue indicates residues found to be involved in heme uptake by *C*. *elegans* HRG-4; orange indicates residues selected for mutagenesis in *L*. *amazonensis* LHR1; light gray boxes indicate predicted transmembrane domains. **B**) Predicted structure of LHR1 with amino acids selected for mutation highlighted. Red = tyrosine; purple = histidine; yellow = arginine; light purple = cysteine.

LHR1 was originally identified by its limited similarity (< 20%) to a heme transporter described in *C*. *elegans*, CeHRG-4, based on its small size and predicted structure of four transmembrane domains [25]. Yuan *et al*. found that in CeHRG-4 a histidine in extracellular loop 2 (His-108) and the C-terminal FARKY motif, a potential heme interacting cluster of basic and aromatic amino acids, were important for heme transport. In the paralogous CeHRG-1 heme transporter, a histidine in transmembrane domain 2 (His-90) and the C-terminal FARKY motif were also found to be involved in heme transport [30]. LHR1 has a His-105 corresponding to His-108 in CeHRG-4 but lacks the FARKY motif, raising the possibility that LHR1 might utilize different amino acids to mediate heme transport. Based on the mutagenesis analysis of CeHRG-4 [30] and results from heme-binding proteins in bacteria [35, 36] we selected nine amino acids for mutation analysis: Tyr-18, His-36, Tyr-64, Tyr-80, His-105, Arg-106, Tyr-129, Tyr-136, and Cys-174 (Fig 1A and 1B). His-105 and Arg-106 were selected because they aligned well with His-108 from CeHRG-4 [30]. All the histidines present in LHR1 (His-16 and His-105) and a few specific tyrosines (Tyr-18, Tyr-64, Tyr-80, Tyr-129, Tyr-136) were selected based on earlier findings showing that these amino acids can be involved in heme coordination [35]. The single cysteine residue (Cys-174) present in LHR1 was also selected because of potential involvement in heme binding and/or intermolecular interactions. All amino acid residues selected for mutation were changed to alanine and the mutant proteins were examined for phenotypes.

Despite extensive efforts, in earlier studies we were unable to generate a homozygous *LHR1* knockout in *L*. *amazonensis*, suggesting that this transporter might be essential for *L*. *amazonensis* survival [25]. Since *LHR1 null* parasites are not viable, direct analyses of mutant LHR1 by genetic complementation was not feasible as a fast and efficient screen. Consequently, we used a heterologous system that would allow us to characterize the ability of the mutant proteins to transport heme without interference from endogenous heme transporters. We took advantage of *S*. *cerevisiae* because it is strongly deficient in the utilization of exogenous heme even when it lacks a functional heme biosynthetic pathway, does not express LHR1 homologs, and engineered reporters and mutant strains are available to evaluate heme transport activity.

**Fig 2:**
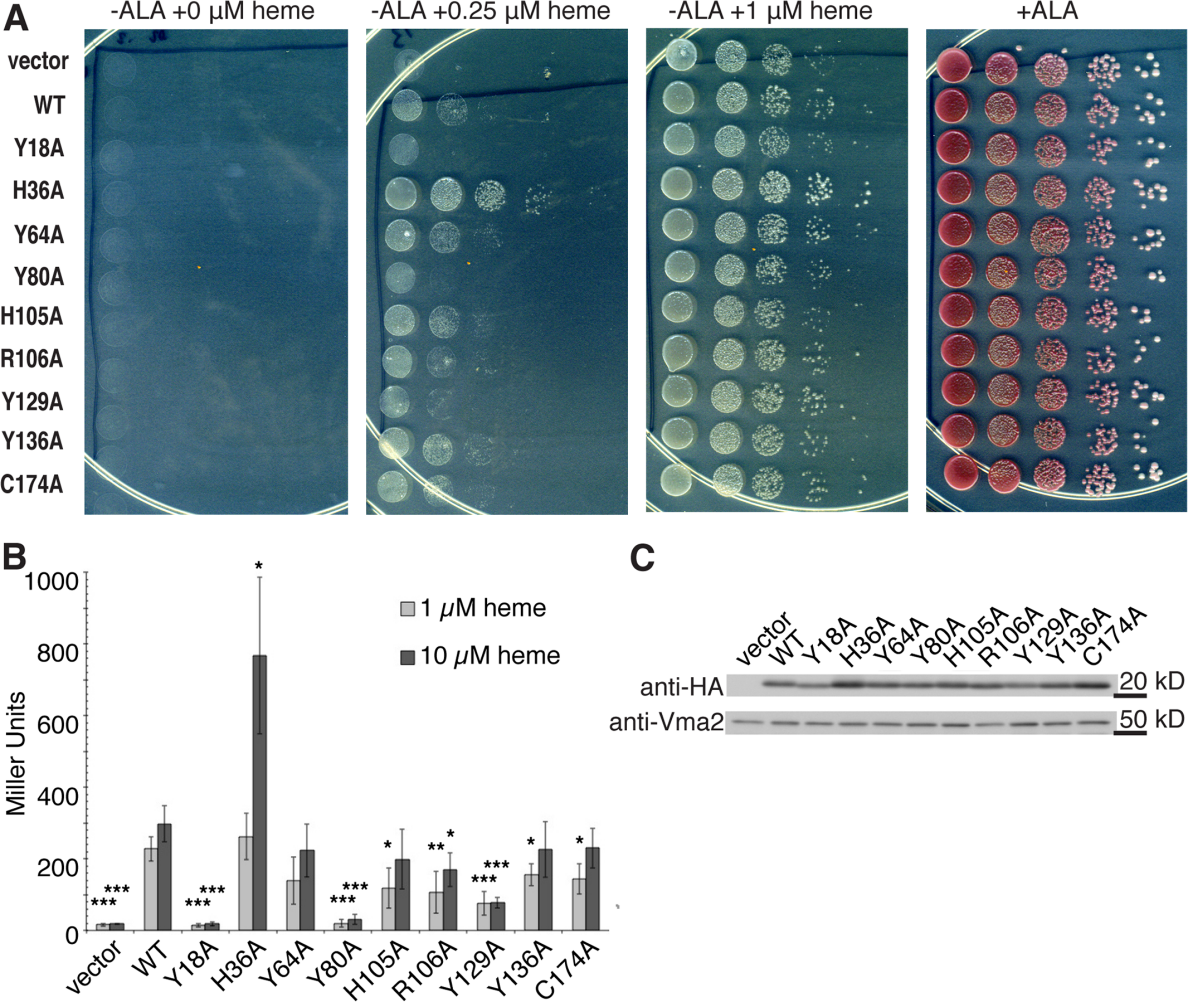
LHR1-mediated growth rescue of a yeast strain defective in heme biosynthesis depends on tyrosine residues. **A**) *S*. *cerevisiae* Δ*hem1* (6D) expressing vector alone or LHR1, WT or mutant proteins, were serially diluted and spotted onto agar plates containing a range of heme concentrations or 250 μM ALA (positive control), incubated at 30°C for four days and imaged. Images are representative of two to four experiments using independent transformants. **B**) Heme-dependent β-galactosidase production in *S*. *cerevisiae* Δ*hem1* (6D) expressing vector alone or LHR1, WT or mutant proteins, grown in 1 or 10 μM heme for 16 h. The results are representative of five-eight experiments performed with independent transformants. Student’s *t*-test: *** p ≤ 0.005, ** p ≤ 0.01, * p ≤ 0.05 compared to WT. **C**) Western blot of *S*. *cerevisiae* Δ*hem1* (6D) expressing vector alone or LHR1, WT or mutant proteins, grown in 10 μM heme.

For the initial experiments we used the *S*. *cerevisiae* Δ*hem1* (6D) strain, which lacks the *HEM1* gene that encodes the first enzyme in the heme biosynthesis pathway, δ-aminolevulinic acid synthase (ALAS) [37]. Δ*hem1* (6D) needs to be supplemented with the product of ALAS, δ-aminolevulinic acid (ALA) or excess heme in order to grow. LHR1 WT and mutant proteins tagged at the C-terminus with an HA epitope were expressed in Δ*hem1* (6D) under an inducible *GAL1* promoter. We used two independent assays, well established in the Δ*hem1* (6D) strain, to assess the ability of the WT or mutant LHR1 proteins to transport heme [37]. First, we assessed the ability of plasmids driving the expression of WT or mutant LHR1 to rescue the growth of Δ*hem1* (6D) on agar plates containing varying heme concentrations (Fig 2A). Cells transformed with pYesDEST52 alone were only able to grow on agar plates when supplemented with ALA or ≥1 μM heme. In contrast, cells transformed with WT *LHR1* were also able to grow at a lower heme concentration. At 0.25 μM heme, yeast cells transformed with Y18A, Y80A, and Y129A *LHR1* mutants showed reduced growth when compared to WT *LHR1*. On the other hand, cells transformed with H36A showed a marked increase in growth (Fig 2A). There was some variation in expression levels between the mutant proteins, with H36A, Y64A and R106A being expressed at higher levels (Fig 2C). H36A showed higher expression levels in several independent experiments, which may account for the marked increase in growth observed in the growth spot assay (Fig 2A). Second, we measured changes in regulatory intracellular pools of heme using β-galactosidase activity from a *CYC*::*lacZ* promoter-reporter fusion. In this system, *lacZ* expression is dependent upon Hap1-5, a transcription complex that binds heme and activates the *CYC1* promoter. Thus, the level of β-galactosidase activity in this assay directly reflects the amount of intracellular heme available to activate the *CYC1* promoter [30]. Cells transformed with pYesDEST52 alone had negligible cytoplasmic heme levels, but an increase in cytoplasmic heme was observed in cells transformed with WT LHR1 (Fig 2B). In contrast, cells expressing the Y18A, Y80A, and Y129A mutant proteins showed a highly significant reduction in their ability to increase cytoplasmic heme levels, at both concentrations of heme tested. Consistent with the results of the spot growth assay, when grown in 10 μM heme cells expressing H36A also showed an increased level of cytoplasmic heme. Some smaller differences were also detected with the additional mutations, but these did not alter cytoplasmic heme levels to an extent that was highly statistically significant (Fig 2B).

**Fig 3:**
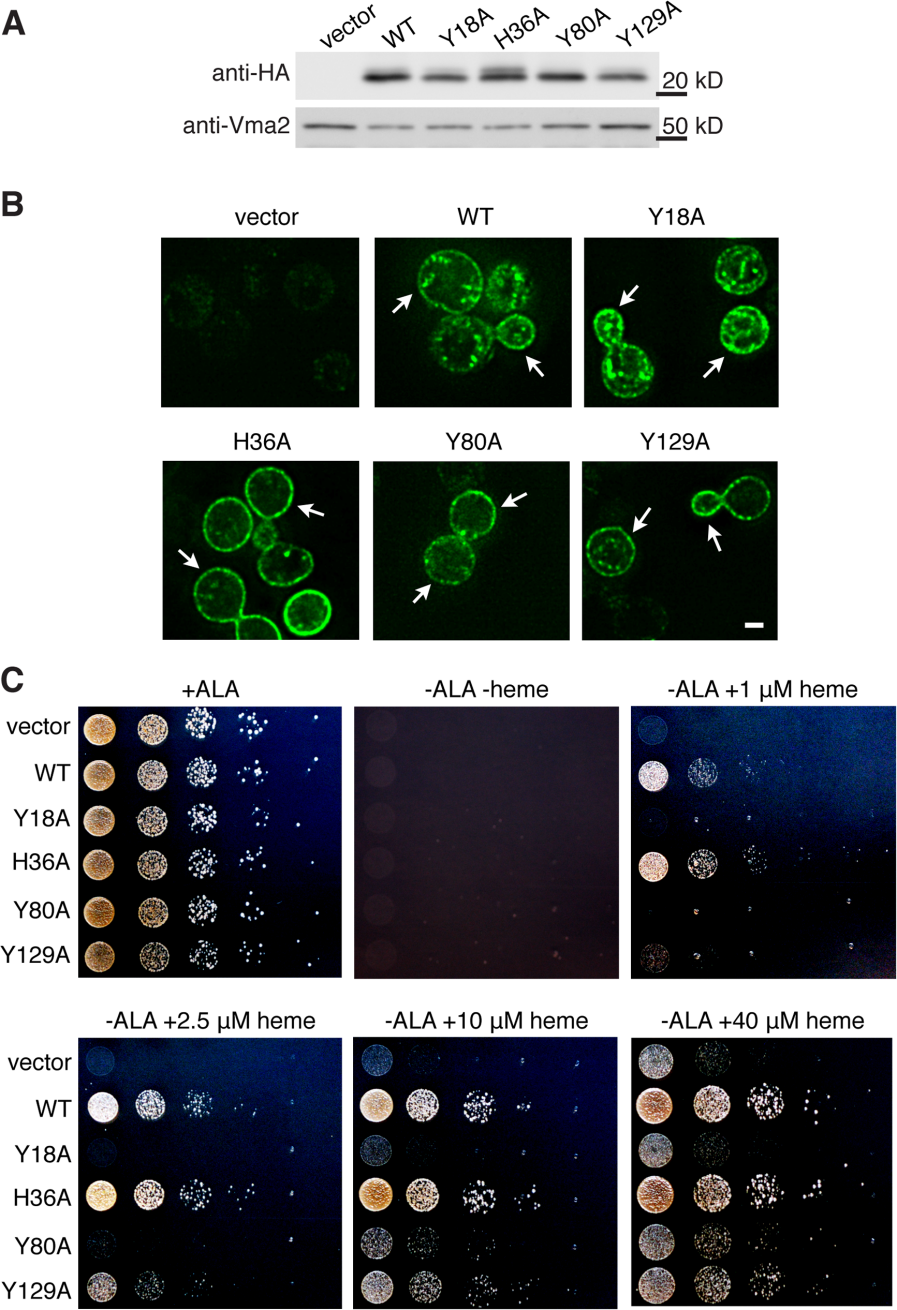
Mutation of Tyr-18, Tyr-80 and Tyr-129 causes varying growth inhibition levels in yeast respiration assays. **A**) Western blot of *S*. *cerevisiae* Δ*hem1* (6D) expressing vector alone or yeast codon-optimized LHR1-HA WT, Y18A, H36A, Y80A or Y129A. **B**) Deconvolution fluorescence images of *S*. *cerevisiae* Δ*hem1* (6D) expressing vector alone or yeast codon-optimized WT or mutant LHR1-HA proteins. The arrows point to the yeast cell plasma membrane. Bar = 2 μm. **C**) *S*. *cerevisiae* Δ*hem1* (6D) expressing vector alone or yeast codon-optimized WT or mutant LHR1-HA proteins were cultivated for 18 h in glycerol/lactate medium, serially diluted and spotted onto agar plates containing glycerol/lactate and supplemented with either 250 μM ALA (positive control) or a range of heme concentrations, incubated at 30°C for four days and imaged. Images are representative of two experiments using independent transformants.

Based on the results of several independent β-galactosidase activity and growth rescue assays performed with the panel of mutants, we selected four mutants for further characterization of their role in heme transport: Y18A, H36A, Y80A, and Y129A. In order to more precisely clarify the role of tyrosines in heme transport under physiological conditions, we generated yeast codon-optimized WT and mutant LHR1 proteins and expressed them in the *S*. *cerevisiae* Δ*hem1* (6D) strain. All proteins were expressed (Fig 3A) and showed a similar localization pattern, that included the plasma membrane (Fig 3B). We then analyzed the wild type and mutant proteins for their ability to transport heme and restore mitochondrial respiration. Yeast mutants defective in oxidative metabolism, such as Δ*hem1* (6D), are unable to grow on glycerol/lactate, a non-fermentable carbon source. Growth of Δ*hem1* (6D) on glycerol/lactate is dependent upon heme availability, which allows cells to metabolize the glycerol/lactate to generate ATP [38]. Consequently, growth on glycerol/lactate reflects the amount of heme that is available within the cell. Yeast expressing vector alone (vector, Fig 3C) were unable to grow even in the presence of 40 μM heme, indicating that the cells cannot utilize exogenous heme for aerobic growth in the absence of a functional heme transporter. Conversely, yeast cells transformed with WT and H36A were able to grow at 1 μM heme (WT and H36A, Fig 3C) indicating that the heme necessary for mitochondrial respiration can be obtained via a fully functional LHR1, and that histidine 36 is not involved in this process. By contrast, Y18A and Y80A showed significantly decreased rescue of growth, indicating impaired heme transport activity (Y18A and Y80A, Fig 3C). Cells expressing Y129A showed an intermediate phenotype, with decreased growth compared to WT but improved growth when compared to Y18A and Y80A (Y129A, Fig 3C). Remarkably, cells expressing Y18A could not grow even in the presence of high levels of exogenous heme, and their growth pattern was comparable to empty vector at all concentrations of heme examined—suggesting that this mutation renders LHR1 non-functional.

**Fig 4:**
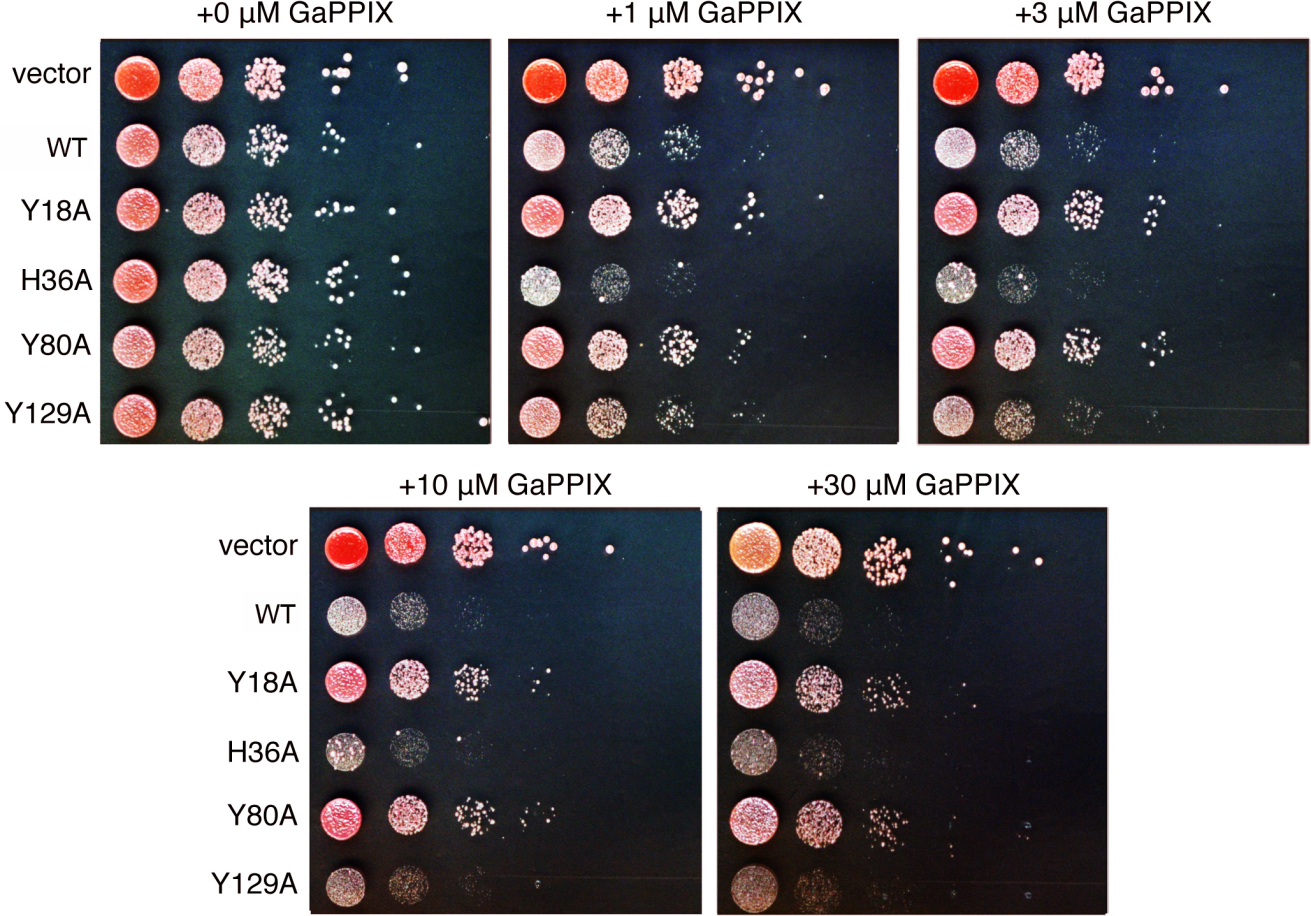
Tyr-18, Tyr-80 and Tyr-129 differentially regulate LHR1-mediated uptake of a toxic heme analog. *S*. *cerevisiae* W303 expressing vector alone or yeast codon-optimized LHR1-HA WT, Y18A, H36A, Y80A or Y129A were serially diluted and spotted onto agar plates with a range of GaPPIX concentrations, incubated at 30°C for three days and imaged. Images are representative of two experiments using independent transformants.

The Y18A, H36A, Y80A, and Y129A mutant LHR1 proteins were further analyzed for their ability to transport heme via a toxicity study in *S*. *cerevisiae* using GaPPIX, a toxic heme analog [39]. Since *S*. *cerevisiae* lack the ability to transport exogenous heme, WT yeast incubated in the presence of GaPPIX show normal growth. On the other hand, cells that express a functional heme transporter are able to import toxic GaPPIX, which results in growth arrest. The W303 *S*. *cerevisiae* strain transformed with empty vector was able to grow at all concentrations of GaPPIX examined, whereas cells expressing WT or H36A showed decreased growth at all concentrations of GaPPIX. This result shows that WT and H36A are functional proteins, capable of importing a toxic heme analog (WT and H36A, Fig 4). The growth of cells expressing Y18A and Y80A was comparable to empty vector except at very high GaPPIX concentrations, indicating that these proteins are unable to transport GaPPIX as efficiently as WT LHR1 (Y18A and Y80A, Fig 4). Y129A showed an intermediate phenotype with decreased growth at all GaPPIX concentrations, but not as severe as Y18A and Y80A. This indicates that Y129A has a decreased ability to transport GaPPIX but is still functional (Y129A, Fig 4). Taken together, these results indicate that Tyr-18 and Tyr-80 are critical for heme transport and that Tyr-129 plays a minor role, whereas His-36 does not appear to be required for heme transport, and is likely to have enhanced heme uptake in our assays due to elevated expression levels.

**Fig 5:**
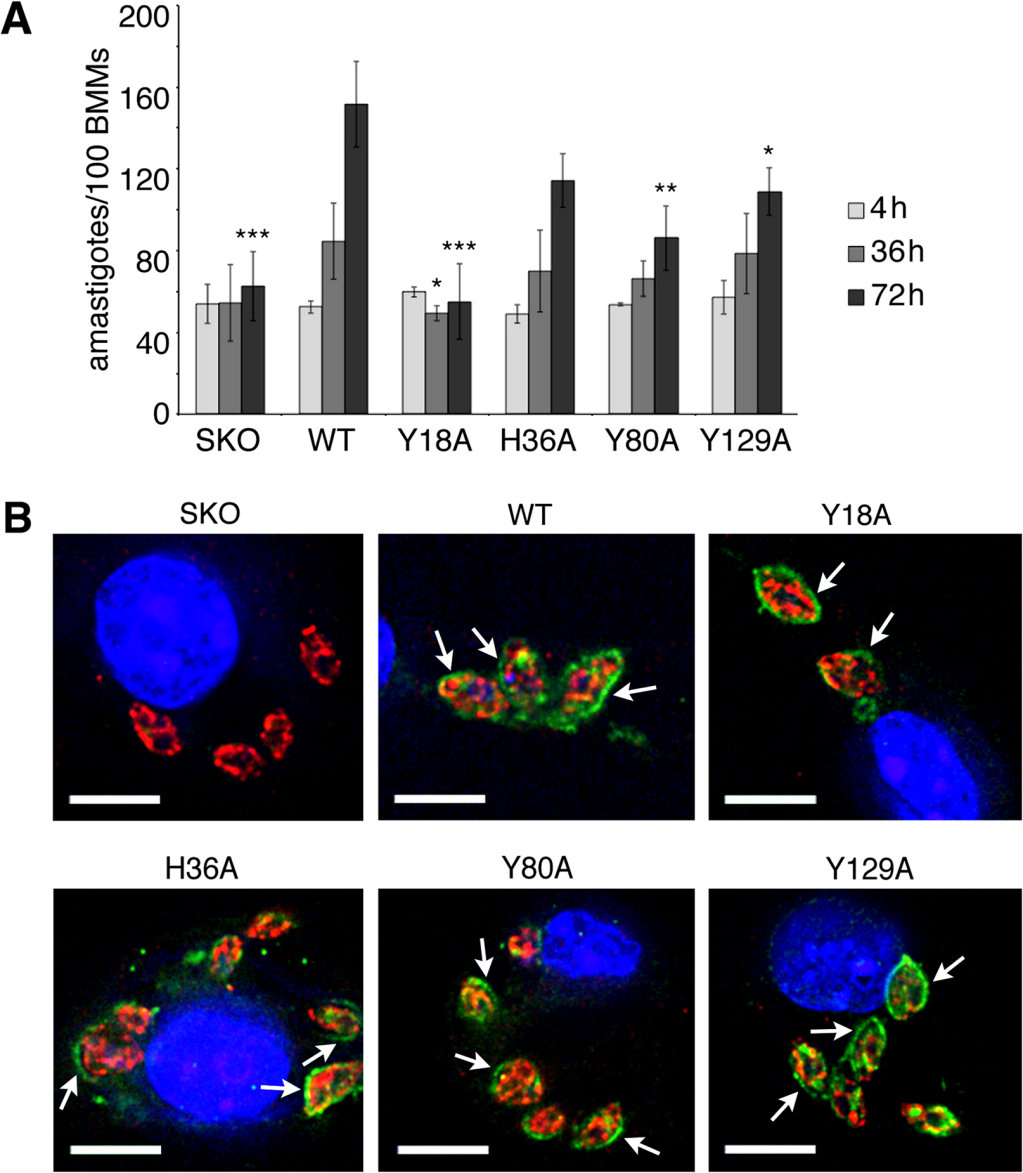
Tyrosine mutations resulting in decreased heme uptake cause equivalent defects in *L*. *amazonensis* intracellular replication. **A**) *L*. *amazonensis* Δ*lhr1*/*LHR1* (SKO) amastigotes expressing WT, Y18A, H36A, Y80A, or Y129A *LHR1*-HA constructs were used to infect mouse BMMs, and intracellular growth was determined microscopically at different time points after infection. The assay was performed in triplicate, and results are representative of three independent experiments. Student’s *t*-test values comparing 72 h time points: WT vs. SKO = 0.005, WT vs. Y18A = 0.004, WT vs. H36A = 0.06, WT vs. Y80A = 0.01, WT vs. Y129A = 0.04; Student’s *t*-test comparing 36 h time point: WT vs. Y18A = 0.03. *** p ≤ 0.005, ** p ≤ 0.01, * p ≤ 0.05. **B**) Deconvolution fluorescence images of infected macrophages showing the localization of wild type and mutant LHR1-HA proteins and the ER marker BiP at 24h post-infection. The arrows point to HA-tagged proteins localized on the plasma membrane of intracellular amastigotes. Images were acquired under similar conditions. Blue = DAPI, red = anti-BiP, green = anti-HA. Scale bar = 6 μm.

With the knowledge that three tyrosines are critical for the function of LHR1 in yeast, we proceeded to verify if these amino acid residues were also functionally important in *L*. *amazonensis*. As mentioned earlier, homozygous knockout mutants of LHR1 in *L*. *amazonensis* are not viable, so we transfected heterozygous Δ*lhr1/LHR1* (SKO) *L*. *amazonensis* with an episomal plasmid expressing LHR1-HA WT, LHR1-HA Y18A, LHR1-HA H36A, LHR1-HA Y80A, or LHR1-HA Y129A. We first examined the non-infective parasite insect forms, promastigotes, for growth defects in culture, and found no differences in growth between SKO+WT and SKO+Y18A, H36A, Y80A, or Y129A (data not shown). Given this lack of phenotype in promastigotes, we examined the infective intracellular amastigote stage, which is responsible for leishmaniasis by replicating within host macrophages. Mouse bone marrow-derived macrophages (BMM) were infected with axenic amastigotes of heterozygous Δ*lhr1/LHR1* (SKO) *L*. *amazonensis* and SKO complemented with WT, Y18A, H36A, Y80A, or Y129A *lhr1*, and intracellular parasites were quantified after 4, 36, and 72 hours post infection (Fig 5A). In agreement with previous observations [26], SKO parasites failed to replicate in BMMs, but growth was partially restored by WT LHR1 with the parasites undergoing approximately two replication cycles during the first 72 h after infection. In contrast, the growth defect of SKO parasites expressing the Y18A mutant was severe, and comparable to SKO parasites without complementation. LHR1 containing the Y80A and Y129A mutations did not rescue growth to the same extent as WT, but grew better than SKO and SKO+Y18A. SKO parasites expressing H36A showed growth comparable to that of SKO expressing WT, with intracellular numbers doubling during the first 72 h (Fig 5A). Immunofluorescence with antibodies against the HA tag showed that the WT and mutant proteins localized to the parasite plasma membrane and occasionally to intracellular compartments, consistent with the previously reported localization of GFP-tagged LHR1 in *L*. *amazonensis* amastigotes [25]. No co-localization was observed with the endoplasmic reticulum marker BiP [40], suggesting that the Y18A, H36A, Y80A and Y129A mutations did not interfere with the proper folding and trafficking of LHR1 in *L*. *amazonensis* (Fig 5B).

**Fig 6:**
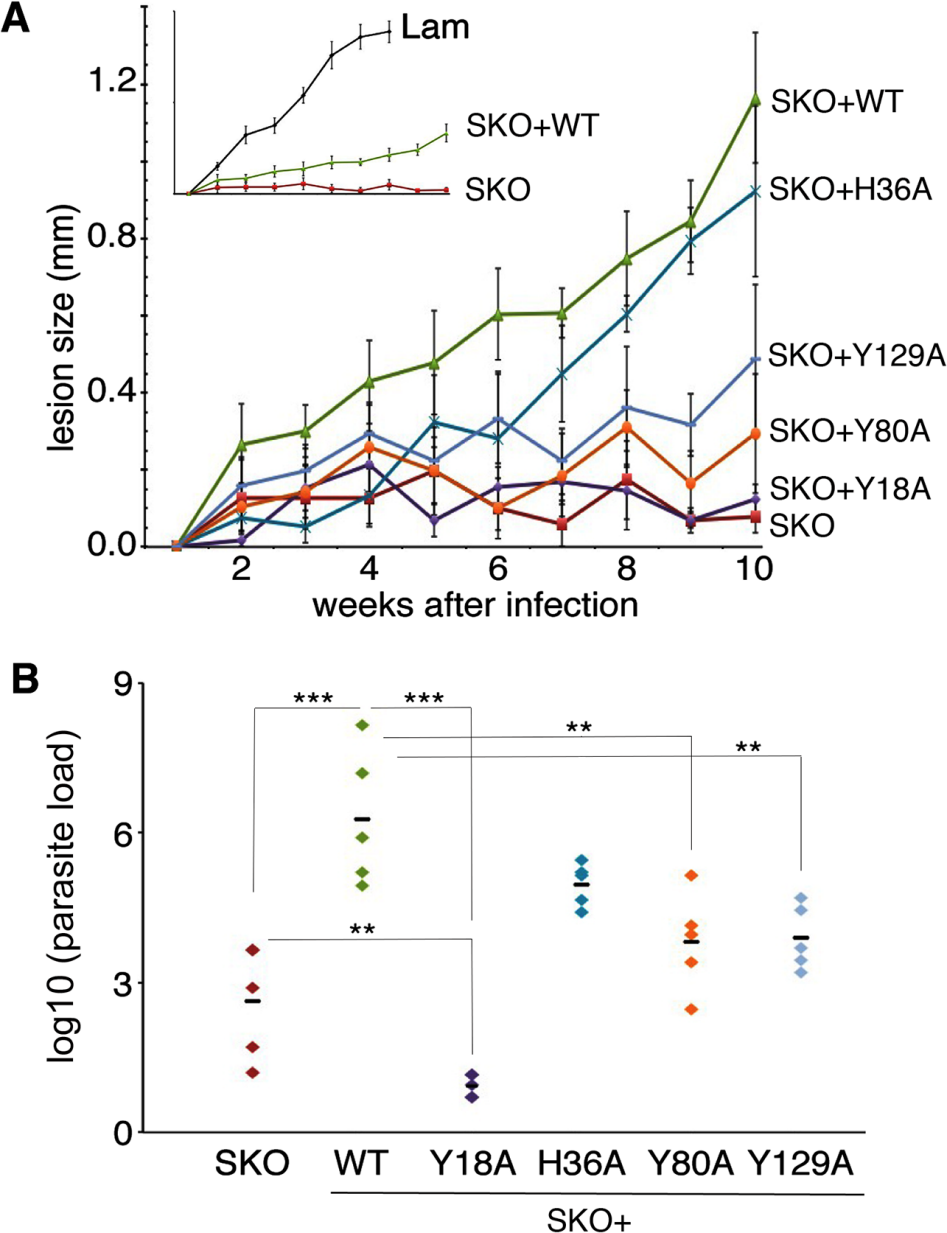
The varying levels of heme uptake inhibition caused by mutations in LHR1 tyrosines 18, 80 and 129 correlate with defects in *L*. *amazonensis* virulence. **A**) Cutaneous lesion growth over a ten-week period in C57BL/6 mice infected with wild type *L*. *amazonensis* (Lam) or Δ*lhr1*/*LHR1* (SKO) expressing the following LHR1 proteins: WT, Y18A, H36A, Y80A, or Y129A. The inset shows the growth of wild type *L*. *amazonensis* (Lam) compared to *L*. *amazonensis* SKO and *L*. *amazonensis* SKO expressing WT-*LHR1* (SKO+WT); axes are the same as the large plot. **B**) Total parasite load in footpad tissue isolated from the mice after ten weeks of infection. Each group had five mice. Student’s t-test values: WT vs. SKO = 0.002; WT vs. Y18A = 0.0005; WT vs. H36A = 0.07; WT vs. Y80A = 0.01; WT vs. Y129A = 0.008; SKO vs. Y18A = 0.01. *** p ≤ 0.005, ** p ≤ 0.01.

To determine the effect of these mutations on the *in vivo* infectivity of *L*. *amazonensis*, axenic amastigotes were injected in the left hind footpad of C57BL/6 mice. Lesion development was followed over a ten-week period, and parasite tissue loads were determined by limiting dilution at week ten. Mice infected with SKO parasites failed to form a lesion, and had low parasite loads at the site of infection (Fig 6A and 6B). Complementation with WT or H36A LHR1 restored lesion development (Fig 6A), albeit not to the same high levels observed after infection with an untransfected *L*. *amazonensis* wild type strain carrying only the two endogenous copies of LHR1 (inset Lam, Fig 6A)—consistent with earlier findings [26]. In agreement with the growth profiles observed in the macrophage infections, SKO parasites expressing Y18A, Y80A, and Y129A LHR1 showed reduced lesion development and smaller parasite tissue loads, when compared to SKO complemented with WT LHR1 (Fig 6A and 6B). Interestingly, the tissue parasite loads observed after complementation with Y18A were lower than what was observed for SKO without complementation (Fig 6B). These findings suggest that the Y18A mutation severely impairs the ability of LHR1 to mediate heme acquisition, to an extent greater than what would be expected if it were a non-functional protein.

## Discussion

Trypanosomatid parasites, which include the human pathogen *Leishmania*, are heme auxotrophs that must acquire heme from the environment to sustain essential metabolic functions [21, 22]. Eukaryotic heme transporters were only recently identified [41, 42] and little is known about structural features of these transporters responsible for heme translocation across membranes. Previous studies identified LHR1, a transmembrane protein with limited similarity to the metazoan HRG heme transporters, which mediates heme uptake in *Leishmania amazonensis* [25]. *L*. *amazonensis* LHR1 null mutants could not be obtained and SKO mutants showed virulence phenotypes that could be partially complemented by episomally expressed LHR1 [26], suggesting that LHR1 was required for parasite virulence. However, it remained unclear whether the virulence defect of LHR1 SKO mutant was directly related to impaired heme uptake, or to indirect consequences of altered protein expression levels. Here we have clarified this issue, by showing that point mutations that impair the heme uptake capacity of LHR1 in yeast also fail to complement virulence in *L*. *amazonensis*. We found that replacement of individual LHR1 transmembrane residues led to different extents of heme transport inhibition in yeast, an effect that was closely mirrored by the impact of these mutations on the ability of LHR1 to restore virulence in *L*. *amazonensis* SKO mutants.

Four mutations of interest emerged from the functional screen we performed in yeast to assess the heme transport capacity of LHR1. Three of these mutations (Y18A, Y80A and Y129A) reduced heme transport function, and one (H36A) appeared to increase heme uptake. However, H36A was consistently expressed in yeast at higher levels when compared to the other mutants, suggesting that the increased heme transport observed may have been a consequence of the presence of more protein in the yeast cells. This conclusion is reinforced by the observation that when expressed in *L*. *amazonensis*, LHR1-H36A showed similar expression and localization and did not enhance virulence, when compared to the WT protein. Remarkably, mutations in LHR1 Tyr-18, Tyr-80 and Tyr-129 behaved very similarly in the heme transport assays in yeast and in virulence assays performed with *L*. *amazonensis*. The Y18A mutation had a severe inhibitory effect on all heme transport assays we performed in yeast, and correspondingly did not rescue the ability of the SKO strain of *L*. *amazonensis* to replicate intracellularly in macrophages or in infected mice. Y80A and particularly the Y129A mutation caused intermediate and highly equivalent phenotypes in the yeast heme uptake and in parasite infectivity assays, directly demonstrating that the efficiency of heme transport by LHR1 is a major determinant of virulence in *L*. *amazonensis*.

Previous studies identified histidine residues that were topologically conserved and essential for heme translocation by the HRG-1-related heme transporters [30]. *L*. *amazonensis* LHR1 contains only two histidine residues, His-36 and His-105. Although LHR1 carrying the H36A mutation was equivalent to WT in its ability to promote heme uptake in yeast, it appeared not to fully restore the *in vivo* virulence of *L*. *amazonensis* SKO. This observation suggests that His-36 may either have a minor role or function together with other residues to affect heme transport, and that the value of His-36 only becomes apparent over time, during an active *Leishmania* infection *in vivo*. In the *C*. *elegans* HRG-4, His-108 was necessary for optimal heme uptake, but the equivalent residue in LHR1 (His-105) showed no phenotype when mutated in our yeast screen and was not pursued further. We can’t rule out the possibility that a mild virulence phenotype might emerge with the His-105 mutation in *Leishmania* infection assays, particularly if combined with other synthetic missense mutations in LHR1, for example H36A. In *C*. *elegans* a histidine residue in the E2 loop, predicted to be located on the extracellular/luminal side of the membrane, was proposed to transfer heme to a histidine in the transmembrane domain 2 of HRG-1, or to a tyrosine in the synonymous position of HRG-4 [30].

In agreement with the known role of tyrosines in heme coordination [43], our study identified functionally important tyrosines within the predicted transmembrane domains 1, 2 and 3 of LHR1. These are positions where the tyrosine residues could potentially interact with heme within the helical membrane segments, as it is being transported across the membrane. Consistent with the low sequence identity of LHR1 with other HRG-1-related proteins, the LHR1 transmembrane residues Y18, Y80 and Y129 are absent in the HRG family. Given the multiple hosts that harbor *Leishmania* (sandflies and mammals), it is conceivable that the unique properties of LHR1 that emerged from our study are related to the need to acquire heme in diverse environments such as the lysosome-like macrophage PV and the insect intestinal tract. The most striking phenotype was observed with the mutation targeting Tyr-18 in TMD1 of LHR1. Not only did parasites carrying the Y18A mutation fail to induce the development of lesions in mouse footpad infections, but the parasite loads subsequently observed at the site of inoculation were lower than what was seen with the non-complemented SKO strain. Plausibly, the Y18A mutation may cause alterations that render the LHR1 permease non-functional through a dominant-negative effect, such as interference with LHR1 multimerization.

Several questions remain regarding the mechanisms involved in heme transport by *Leishmania*. LHR1 is a small protein of only 175 amino acids with four predicted transmembrane domains that probably does not have the size necessary to form a channel large enough to accommodate heme. It is still unknown whether LHR1 functions as a monomer or an oligomer, and what are the implications of a potential oligomerization for the process of heme acquisition by these parasites. It will also be important to understand how LHR1 obtains the energy to transport heme against a concentration gradient. LHR1 doesn’t contain an ATP-binding cassette, but this doesn’t rule out the possibility that heme transport by LHR1 may be coupled to an ATP-dependent process. Indeed, studies have shown that the human HRG1 transporter interacts with the vacuolar V-type ATPase [44]. Heme uptake in *T*. *cruzi* has been reported to be ATP dependent [45], but whether this represents a process mediated by the *T*. *cruzi* LHR1 homolog or an alternative heme acquisition pathway via hemoglobin breakdown [46] remains to be determined.

In addition to identifying unique amino acids critical for LHR1 function, our study reveals for the first time that there is a strong correlation between the capacity of LHR1 to transport heme across cellular membranes and the ability of *L*. *amazonensis* to establish infections in a mammalian host. This new information reinforces the possibility that small molecule antagonists selectively targeting heme transport by LHR1 might be effective for controlling *Leishmania* infections—fulfilling the urgent need for better options to combat these serious human diseases. Since *LHR1* is well conserved across the Trypanosomatidae family [25], new therapeutic agents targeting this transporter could have an impact on a number of additional diseases that affect millions of people, including Chagas’ disease in Latin America and sleeping sickness in Africa.
